# Therapeutic plasma exchange in amatoxin associated acute liver failure–results from the multi-center Amanita-PEX study

**DOI:** 10.1186/s13054-025-05560-y

**Published:** 2025-10-30

**Authors:** Klaus Stahl, Bahar Nalbant, Thorben Pape, Isaure Breteau, Valentin Coirier, Filipe S. Cardoso, Jubi de Haan, Maciej K. Janik, Jan-Christian Wasmuth, João Madaleno, Uta Merle, Josephine Frohme, Phil-Robin Tepasse, Martina Müller, Karsten Große, Alexandra Linke, Nikola Mareljic, Fin Stolze Larsen, Gérladine Dahlqvist, Mirjam Kolev, Marie Schulze, Katharina Willuweit, Petra Janke-Maier, Felix Dondorf, Óscar M. Fierro-Angulo, Anja Geerts, David Toapanta, Camille Dejean, Mohamed Alharthi, Enric Reverter, Heiko Schenk, Sarah Raevens, Ricardo Ulises Macías-Rodríguez, Falk Rauchfuß, Christoph P. Berg, Hartmut Schmidt, Andreas Geier, Nasser Semmo, Nicolas Lanthier, Peter N. Bjerring, Christian M. Lange, Martina Sterneck, Tony Bruns, Stephan Schmid, Dominik van de Loo, Münevver Demir, Tobias Boettler, Catarina Borges, Jacob Nattermann, Karolina Wronka, Caroline M. den Hoed, Hugo P. Marques, Florent Artru, Eric Levesque, Heiner Wedemeyer, Alejandro Campos-Murguia, Richard Taubert

**Affiliations:** 1 Department of Gastroenterology, Hepatology, Infectious Diseases and Endocrinology, Hannover Medical School, Carl-Neuberg Str. 1, Hannover, 30625 Germany; 2 Department of Respiratory Medicine and Infectious Diseases, and German Centre of Lung Research (DZL), Hannover Medical School, Hannover, Germany; 3 Department of Anesthesia and Intensive Care, University Hospitals of Tours, Tours, France; 4 Liver Department, Médecine Intensive Réanimation, Rennes University Hospital, Rennes, France; 5 Curry Cabral Hospital, Nova Medical School, Lisbon, Portugal; 6 Department of Gastroenterology and Hepatology, Erasmus MC Transplant Institute, Erasmus University Medical Center, Rotterdam, The Netherlands; 7 Liver and Internal Medicine Unit, Medical University of Warsaw, Warsaw, Poland; 8 Department of Internal Medicine I, German Center for Infection Research (DZIF), University of Bonn, Bonn, Germany; 9 Liver Disease Unit - Internal Medicine Department and Adult Liver Transplant Unit, Hospitais da Universidade de Coimbra, Unidade Local de Saúde de Coimbra, Coimbra, Portugal; 10 Medical Faculty, Department of Internal Medicine IV, Heidelberg University, University Hospital, Heidelberg, Germany; 11 Department of Hepatology and Gastroenterology, Campus Virchow-Klinikum and Campus Charité Mitte, Charité - Universitätsmedizin Berlin, Berlin, Germany; 12 Department of Medicine B for Gastroenterology, Hepatology, Endocrinology and Clinical Infectiology, University Hospital Muenster, Muenster, Germany; 13 Department of Internal Medicine I, Gastroenterology, Hepatology, Endocrinology, Rheumatology, and Infectious Diseases, University Hospital Regensburg, Regensburg, Germany; 14 Department of Internal Medicine III, University Hospital RWTH Aachen, Aachen, Germany; 15 Department of Medicine, University Medical Centre Hamburg-Eppendorf, Hamburg, Germany; 16 Department of Medicine II, University Hospital, Ludwig Maximilian University of Munich, Munich, Germany; 17 Department of Hepatology and Gastroenterology, Rigshospitalet, Copenhagen University Hospitalet, Copenhagen, Denmark; 18 Service d’Hépato-gastroentérologie, Cliniques universitaires Saint-Luc, UCLouvain, Brussels, Belgium; 19 Department of Visceral Surgery and Medicine, Bern University Hospital, Inselspital, University of Bern, Bern, Switzerland; 20 Department of Internal Medicine II, Hepatology, University Hospital of Würzburg, Würzburg, Germany; 21 Department of Gastroenterology, Hepatology and Transplantational Medicine University Hospital Essen, Faculty of Medicine, University of Duisburg-Essen, Essen, Germany; 22 Department of Gastroenterology, Gastrointestinal Onkology, Hepatology, Infektious Diseases,and Geriatrics, University of Tuebingen, Tuebingen, Germany; 23 Department of General, Visceral and Vascular Surgery, Jena University Hospital, Jena, Germany; 24 Division of Hepatology, Instituto Nacional de Ciencias Médicas y Nutrición Salvador Zubirán, Mexico City, Mexico; 25 Department of Gastroenterology & Hepatology, Ghent University Hospital, Ghent, Belgium; 26 Liver Unit, IDIBAPS, and CIBEREHD, Liver ICU, Hospital Clinic, University of Barcelona, Barcelona, Spain; 27 Department of Medicine II, Faculty of Medicine, Medical Center - University of Freiburg, Freiburg, Germany; 28 Apheresis Unit - Blood and Transfusion Medicine Department, Hospitais da Universidade de Coimbra, Unidade Local de Saúde de Coimbra, Coimbra, Portugal; 29 European Reference Network on Hepatological Diseases (ERN RARE-LIVER), Hamburg, Germany; 30 Institute of Intensive Care Medicine, University Hospital Zurich, Zurich, Switzerland; 31 Department of Nephrology and Hypertension, Hannover Medical School, Hannover, Germany; 32 Department of Intensive Care, Erasmus University Medical Center, Rotterdam, The Netherlands

**Keywords:** Liver failure, Mushroom poisoning, Plasma exchange, Liver transplantation, Amanita

## Abstract

**Background:**

Amatoxin-related acute liver failure (AT-ALF) carries high mortality without liver transplantation (LTX). While therapeutic plasma exchange (PEX) might improve LTX-free survival in other ALF cases, its role in AT-ALF is unclear. Clinical practice varies, and, given the rarity of this ALF entity, the feasibility of conducting a randomized controlled trial to investigate PEX in AT-ALF is more or less impossible.

**Methods:**

The Amanita-PEX study is a multi-center, international, retrospective study analyzing patients with AT-ALF from 2013 to 2024. The primary outcome was 28-day LTX-free survival (composite endpoint: death or LTX) after ALF diagnosis.

**Results:**

The study included 111 patients from 25 centers: 82 received standard-of-care (SOC), and 29 received at least one PEX-session. PEX and SOC-groups were comparable at baseline, but 76% of PEX- vs. 58% of SOC-patients developed hepatic-encephalopathy (HE) grade ≥ 2 (*p* = 0.021). While the primary outcome of 28-day LTX-free survival in all patients was not different between the SOC and PEX-groups, in the subgroup of patients with maximal HE grade ≥ 2, LTX-free survival was 19.1% (*n* = 8/42) in the SOC group, while it was 36.4% (*n* = 8/22) in patients receiving adjunctive PEX (Gehan-Breslow-Wilcoxon-*p* = 0.041, Log-Rank-*p* = 0.060). PEX was independently associated with reduced risk of the combined endpoint death or liver transplantation within 28 days from inclusion in patients with HE grade ≥ 2 (HR 0.37, 95%-CI 0.19–0.73, *p* = 0.004). After propensity-score-matching, LTX-free survival was 28% in the SOC- and 52% in the PEX group (Gehan-Breslow-*p* = 0.036; Log-Rank-*p* = 0.035).

**Conclusions:**

In this real-world study, adjunctive use of PEX was associated with increased LTX-free-survival in patients with AT-ALF and HE grade ≥ 2.

**Supplementary Information:**

The online version contains supplementary material available at 10.1186/s13054-025-05560-y.

## Highlights

- Acute liver failure due to ingestion of mushrooms containing amatoxins has a poor prognosis when higher grade hepatic encephalopathy develops.
- Adjunctive use of therapeutic plasma exchange was independently associated with increased liver transplant-free-survival in patients with amatoxin associated acute liver failure and maximum hepatic encephalopathy grade ≥ 2.
- Therapeutic plasma exchange was not associated with increased liver transplant-free-survival in patients with hepatic encephalopathy grade 1 and did not improve overall-survival or other secondary endpoints such as shorter length of hospital stay or lower incidence of acute kidney injury, need for renal-replacement therapy, invasive ventilation or vasopressor support.

**Supplementary Information:**

The online version contains supplementary material available at 10.1186/s13054-025-05560-y.

## Impact and Implications

Therapeutic plasma exchange is frequently used in the management of patients with acute liver failure but its effect on improving liver transplant-free-survival has recently been questioned. Amatoxin-associated acute liver failure is a rare entity of acute liver failure and solid data concerning clinical outcomes are scarce. This multi-national, multi-center, real-world, retrospective study suggests that therapeutic plasma exchange is significantly associated with improved liver transplant-free survival only in patients with amatoxin-associated acute liver failure and higher-grade hepatic encephalopathy. These results might help to guide the future use of therapeutic plasma exchange in this specific patient population.

**Graphical abstract:**

**Supplementary Information:**

The online version contains supplementary material available at 10.1186/s13054-025-05560-y.

## Graphical abstract

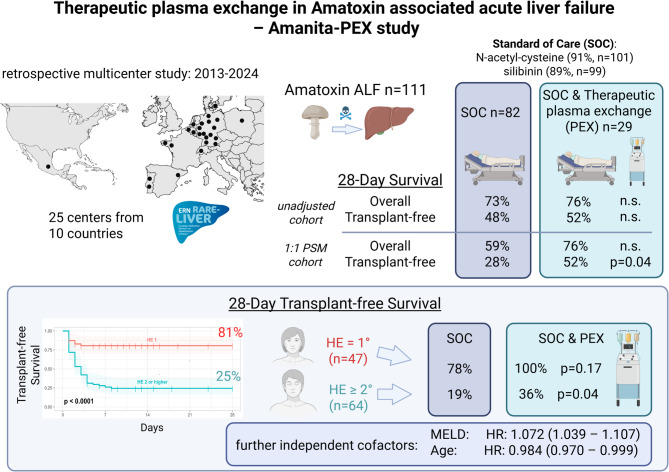

## Introduction

Acute liver failure (ALF) is a severe and complex syndrome that results from massive hepatocellular necrosis in patients without previous cirrhosis and is defined by the acute development of both synthetic failure and hepatic encephalopathy (HE) [1]. Despite different potential etiologies initially triggering the onset of ALF, ALF then takes a common progressive disease course characterized by a dysregulated systemic inflammatory response syndrome (SIRS) and subsequent multiple organ failure (MOF) [2]. This dysregulated immune response is triggered by the release of damage-associated molecular patterns (DAMPs) resulting from hepatocyte cell death and subsequent production of pro-inflammatory cytokines [3, 4]. In line with this hypothesis, several studies have shown that the presence of SIRS is associated with a worsening of HE or MOF and a particularly poor prognosis in ALF [3, 5, 6].

Therapeutic plasma exchange (PEX) with fresh frozen plasma (FFP) used as replacement fluid combines two treatment principles in a single intervention: [1] removal of deleterious DAMPs and cytokines thus modulating the pathological overwhelming immune response that counts responsible for the associated MOF and [2] replacement of the excretory and metabolic functions of the failing liver via supplementation of the lacking proteins contained in healthy donor plasma [7]. Several studies investigating PEX in ALF demonstrated safety and positive effects on multiple clinical parameters such as hemodynamic stability and severity of HE [8–13]. In 2016, a multi-center randomized controlled trial (RCT) compared standard medical therapy only with medical therapy and additional high-volume PEX demonstrating improved overall hospital survival and liver-transplant (LTX)-free survival in patients with ALF [7]. Subsequent smaller studies suggested preserved efficacy of PEX in ALF despite employing normal exchange volume regimens [8, 13, 14]. However, a recent large multi-center retrospective propensity-score-matched study, investigating PEX in ALF under real world conditions, demonstrated no improvement of LTX-free survival by the additional use of PEX [15]. Heterogeneity with respect to employed study designs, including different ALF etiologies, timing and dosing of PEX treatment as well as baseline degree of disease severity were discussed as potential factors explaining these conflicting results [16]. The relevance of universal use of PEX in ALF remains controversial, and recent data underscore the need to identify specific ALF patient populations who might benefit most from PEX.

ALF due to ingestion of mushrooms containing amatoxins (AT-ALF) is a rare cause of ALF and is associated with a high mortality rate in the absence of LTX [17, 18]. While oral detoxification by repeated activated charcoal administration and parenteral infusion of silibinin, a water soluble silymarin derivate, competing with amatoxins for transmembrane transport thus inhibiting the penetration of amanitin into hepatocytes, have evolved as effective standard therapies in the early phase of intoxication and hepatic injury, no specific therapeutic options have been described so far, beyond general critical care support and administration of n-acetyl-cysteine [19] in patients progressing to manifest as hyperacute ALF [20]. While PEX has been incorporated as standard treatment for ALF in many centers following the results of Larsen et al. [7] and positive guidelines recommendations for its use in ALF [1, 21, 22], it nevertheless remains unclear whether PEX may also enhance LTX-free survival in AT-ALF. Not one patient with AT-ALF was included in the key RCTs investigating PEX in ALF [7, 14] and in the retrospective multicenter study from UK [15].

Additionally, there is considerable variation in the clinical practice of utilizing PEX in AT-ALF across different centers. Given the rarity of this specific ALF entity, the feasibility of conducting a randomized controlled trial to investigate the use of PEX in AT-ALF remains uncertain if not elusive.

The aim of this multicenter, multi-national, real-world investigation (Amanita-PEX study) was to record and compare the LTX-free survival between patients who received standard of care (SOC) and PEX and those who received only SOC in AT-ALF.

## Materials and methods

### Study design and setting

We conducted a retrospective, multi-national, multicenter, cohort study of patients from 25 tertiary LTX centers in Germany (Hannover, Aachen, Munich, Berlin, Muenster, Hamburg, Heidelberg, Regensburg, Bonn, Wuerzburg, Essen, Tuebingen, Jena), Spain (Barcelona), Portugal (Lisbon, Coimbra), France (Rennes, Tours), the Netherlands (Rotterdam), Denmark (Copenhagen), Belgium (Ghent, Brussels), Poland (Warsaw), Switzerland (Bern) and Mexico (Mexico City) (Amanita-PEX study, registered as NCT06187220). This report presents the findings for the adult cohort. The study group was initiated and organized within the European Reference Network on Hepatological Diseases (ERN RARE-LIVER). The study was initiated in autumn 2023. The recruitment phase was set to 10 years to reduce the bias of historic cohorts. The landmark RCT by Larsen was published in 2016 [7] and the landmark RCT showing an effect of NAC in 2009 [23]. After one year, we decided to stop recruitment with the deadline end of 2024, because we could not recruit any additional centers of interest.

### Inclusion and exclusion criteria

Diagnosis of ALF was made according to the European Association for the Study of the Liver (EASL) criteria in patients with severe acute liver injury, indicated by the presence of both coagulopathy (international normalized ratio [INR] > 1.5) and manifest HE, without pre-existing chronic advanced liver disease, following laboratory confirmed Amatoxin ingestion [1]. Patients with severe amatoxin-associated hepatitis without fulfilling ALF criteria were excluded from final analysis. The severity of HE was based on the West Haven criteria [22]. Consecutive patients with AT-ALF aged ≥ 18 years were included into the study between October 2013 and October 2024. Patients with acute-on chronic liver failure (ACLF), acute liver injury without encephalopathy and patients with a history of previous LTX were excluded. We compared patients who underwent at least one session of PEX during hospital stay to those who only received SOC. Patients received full organ failure support, which included renal replacement therapy, invasive ventilation and vasopressor support. Patient requirement and eligibility for LTX was assessed by interdisciplinary in-house LTX teams and need for LTX was defined by the King’s-College poor prognostic criteria [1]. Patients with ALF were assigned to the highest waiting list prioritization for graft allocation. The decision to perform adjunctive PEX in addition to SOC was based on the clinical judgement of the treating clinicians and existing specific in-house standards (. Decisions for performing PEX were based on a combination of illness severity at time of presentation, progressive deterioration despite SOC or in patients with contraindications to transplantation as a bridge-to-recovery strategy. PEX dose, used PEX device and number of performed PEX sessions as well as type of adjunct anticoagulation were used according to local protocols, which were not standardized across different centers.

### Data collection

Data were collected retrospectively from medical records as well as patient data monitoring systems (PDMS) and were anonymized before sharing and final analysis. Baseline demographics, clinical and biochemical characteristics were recorded at the first day of fulfillment of definite ALF criteria (e.g. manifest HE and INR > 1.5). Baseline prognostic scores including the MELD score [24] and the Sequential organ failure assessment (SOFA) score [25] were calculated as described elsewhere. Procedural details of performed PEX e.g. time since hospital admission, number of sessions, volume exchanged and type of exchange fluid were recorded.

### Outcome parameters

Outcome parameters were pre-defined as outlined in clinicaltrials.gov (NCT06187220) in 12/2023. The primary outcome was LTX-free survival within the first 28 days after fulfillment of ALF criteria (HE and INR > 1.5). The primary endpoint, 28-day LTX-free survival, was a composite endpoint of the two endpoints death or liver transplantation within the first 28 days following study inclusion. Secondary endpoints were overall survival (OS), need for invasive ventilation, need for vasopressor therapy and need for renal replacement therapy (RRT) until day 28.

### Statistical analysis

Categorical variables are shown as numbers (n) and percentages (%). Unless indicated otherwise, continuous variables are shown as median and 25 − 75% quartiles. Variables were checked for normal distribution using the D’Agostino-Pearson omnibus normality test and the Shapiro-Wilk normality test. For comparisons, two-sided paired t-test, Mann–Whitney U test, or chi-square test were used accordingly. The primary endpoint, LTX-free survival until day 28, was visualized by Kaplan-Meier graphs, and between-group differences were analyzed by Gehan-Breslow-Wilcoxon and Log-rank tests. Gehan-Breslow-Wilcoxon p values were used preferentially as the majority of events occurred early within the first seven days following study inclusion. Multivariate Cox proportional hazard regression and multivariate competing risk regression models were computed to analyze the influence of adjunctive PEX on the primary endpoint. The following fixed covariables were used for the multivariate Cox regression and competing risk regression models (R packages *survival*, *cmprsk* and *finalfit*): Sex, age, MELD-Score at baseline, maximum HE greater than grade 1 during hospital stay, as well as use of adjunctive PEX. Propensity score matching (PSM) was conducted with a Match It Package [26]. Matching was done using the nearest available neighbor in a 1:1 ratio. A standardized mean difference of less than 0.1 was considered as balanced matching.

All reported p-values are two-sided unless indicated otherwise; p-values < 0.05 were considered statistically significant. SPSS Statistics Version 25 (SPSS Inc., Chicago, IL, USA) and the R environment for statistical computing version 4.1.2 (R Foundation for Statistical Computing, Vienna, Austria) were used for data analysis and graph generation.

### Ethics

The study was conducted according to the principles of the Declaration of Helsinki and was approved by the Institutional Review Board of the Hannover Medical School (No 11488_BO_K_2024). All data was fully anonymized before sharing and further analysis.

## Results

### Patients

**Fig. 1:**
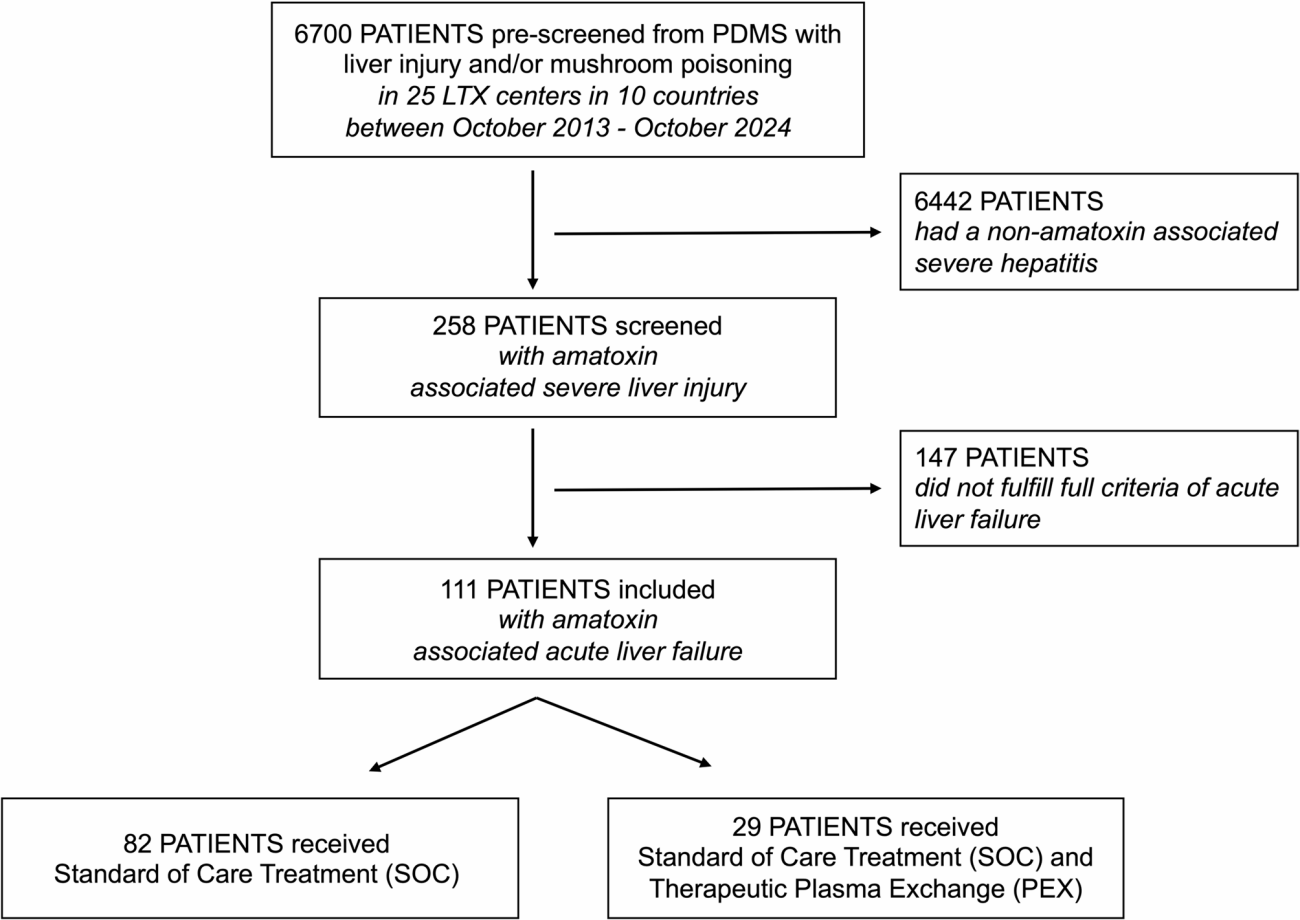
Flow chart of study participants. Shown are pre-screening, screening, inclusion and analysis of patients. Included were patients with the diagnosis of acute liver failure following laboratory confirmed Amatoxin ingestion. The diagnosis of acute liver failure was made according to the European Association for the Study of the Liver (EASL) criteria in patients with severe acute liver injury, indicated by the presence of both coagulopathy (international normalized ratio [INR] > 1.5) and manifest hepatic encephalopathy, without pre-existing chronic advanced liver disease. The study compared standard of care (SOC) with SOC + therapeutic plasma exchange (PEX). This was a retrospective, multicenter, multi-national, real-world study

Between October 2013 and October 2024, 6700 patients were pre-screened using institutional specific PDMS or medical controlling algorithms, of which 258 patients were then screened with severe amatoxin-associated hepatitis and 147 were excluded for not fulfilling full ALF criteria. For final analysis, a total of 111 patients with AT-ALF were included from the following 25 tertiary LTX centers from 10 countries: Germany (*n* = 48: Hannover 15, Bonn 5, Berlin 4, Heidelberg 4, Muenster 4, Regensburg 4, Aachen 3, Hamburg 3, Munich 2, Essen 1, Jena 1, Tuebingen 1, Wuerzburg 1), France (*n* = 30: Tours 18, Rennes 12), Portugal (*n* = 11: Lisbon 7, Coimbra 4), Netherlands (*n* = 7: Rotterdam 7), Poland (*n* = 6: Warsaw 6), Belgium (*n* = 3: Brussels 2, Ghent 1), Switzerland (*n* = 2: Bern 2), Denmark (*n* = 2: Copenhagen 2), Spain (*n* = 1: Barcelona 1), Mexico (*n* = 1: Mexico City 1) (Fig. 1). Further study centers had no amanita ALF cases in the study period from 2013 to 2024: King’s College London/UK, Padua/Italy, Toronto and Edmonton/Canada, Genf/Switzerland and Hôpital Paul Brousse Villejuif/France. A most recent retrospective multicenter study with all UK transplant centers between 2013 and 2021 did not recruit any amanita ALF and could therefore not contribute to this study [15].

Of the 111 patients included, 82 (74%) received SOC treatment and 29 (26%) patients received at least one session of additional PEX. PEX was applied in 10 of the 25 (40%) study centers and in six of the 10 (60%) countries. Table 1 displays the baseline clinical characteristics of all included patients as well as SOC and PEX subgroups, respectively. Median (Interquartile Range [IQR]) age of patients was 54 (42–60) years and 58% (*n* = 64) were female. Median (IQR) time difference from ingestion to hospital admission was 2 (1–3) days. At the first day of fulfillment of ALF criteria (= baseline), high concentrations of Alanine amino-transferase (ALT) and Aspartate amino-transferase (AST) were observed. Median (IQR) bilirubin was 69 (36–105) µmol/l. Blood lactate concentrations were increased in almost all patients as were concentrations of ammonia. Almost half of the patients already had a HE of greater than grade 2 at the first day of ALF. The majority of patients had concomitant acute kidney injury (AKI) (60%, *n* = 67) with 29% (*n* = 32) requiring RRT. RRT was started after initiation of PEX in all but one center. Invasive ventilation was needed in 21% (*n* = 23) and vasopressor support in 25% (*n* = 27). At baseline, the median (IQR) MELD- and SOFA-Scores were 31 (24–39) and 6 (3–9) points, respectively. Almost all patients received supportive therapy with intravenous application of N-acetyl-cysteine (91%, *n* = 101) and silibinin (89%, *n* = 99), with a median time difference from ingestion to silibinin treatment spanning 2 (1–3) days.

No significant differences in neither demographic or clinical parameters nor overall clinical disease severity were found between the SOC and PEX groups at baseline. The use of N-acetyl-cysteine (*p* = 0.644) and silibinin (*p* = 0.43) was also not different between both groups. The MELD Score was numerically higher in the PEX group (36 (30–40) vs. 30 (23–39) points, *p* = 0.068) without reaching statistical significance.

**Table 1:** Demographic and clinical characteristics at study inclusion

Category	All *n* = 111	SOC *n* = 82	PEX *n* = 29	*p*
Age - years (median, [IQR])	54 [42–60]	55 [41–65]	62 [49–69]	0.357
Sex - n (%)				0.234
Female	64 (57.7)	50 (61)	14 (48.3)	
Male	47 (42.3)	32 (39)	15 (51.7)	
BMI - kg/m^2^ (median, [IQR])	25.7 [22.2–31.1]	25.5 [22.9–29]	25.7 [23.3–28.9]	0.193
Time difference ingestion to hospital admission - days (median, [IQR])	2 [1–3]	2 [1–3]	2 [1–3]	0.944
AST - U/l (median, [IQR])	3367 [1580–5864]	3473 [2348–5864]	2596 [1373–6364]	0.830
ALT - U/l (median, [IQR])	4671 [2217–7817]	4899 [2438–7515]	4350 [1980–8456]	0.835
INR (median, [IQR])	3.96 [2.4–6.5]	3.79 [2.05–6.28]	4.17 [2.71–6.77]	0.409
Bilirubin - µmol/l (median, [IQR])	69 [36–105]	62 [32–111]	80 [52–99]	0.8
Creatinine - µmol/l (median, [IQR])	103 [67–192]	105 [68–189]	83 [53–196]	0.496
Lactate - mmol/l (median, [IQR])	4 [2.5–7.6]	3.8 [2.3–9.6]	4.2 [2.9–7.3]	0.485
Ammonia - µmol/l (median, [IQR])	83 [56–119]	77 [54–119]	89 [58–129]	0.724
HE - n (%)	111 (100)	82 (100)	29 (100)	1
HE grade > 1 - n (%)	50 (45.5)	37 (45.7)	13 (44.8)	0.937
AKI - n (%)	67 (60.4)	49 (59.8)	18 (62.1)	0.827
RRT - n (%)	32 (28.8)	21 (25.6)	11 (37.9)	0.208
Invasive ventilation - n (%)	23 (20.7)	19 (23.2)	4 (13.8)	0.284
Oxygenation index (pO_2_/FiO_2_) - mmHg (median, [IQR])	395 [333–451]			0.776
Vasopressors - n (%)	27 (24.5)	21 (25.9)	6 (20.7)	0.574
SOFA score - points (median, [IQR])	6 [3–9]	6 [3–9]	5 [3–9]	0.609
MELD score - points (median, [IQR])	31 [24–39]	30 [23–39]	36 [30–40]	0.068
N-acetyl-cysteine therapy - n (%)	101 (91)	74 (90.2)	27 (93.1)	0.644
Silibinin therapy - n (%)	99 (89.2)	72 (87.8)	27 (93.1)	0.430
Time difference ingestion to silibinin treatment - days (median, [IQR])	2 [1–3]	2 [1–3]	2 [1–3]	0.562

AKI – Acute kidney injury, ALT – Alanine amino-transferase, AST – Aspartate amino-transferase, BMI – body mass index, HE – Hepatic encephalopathy, INR – International normalized ratio, MELD – Model of End-stage liver disease, IPV – Individual patient´s plasma volume, PEX – Therapeutic Plasma Exchange, RRT – Renal replacement therapy, SOFA - Sequential Organ Failure Assessment

Given are demographic and clinical characteristics of the whole patient cohort (n=111), as well as the standard of care (SOC) and plasma exchange (PEX) subgroups at the time of first fulfillment of full criteria for acute liver failure (ALF). Values are presented as median (25% to 75% interquartile-range [IQR]) or if categorical as numbers and percentages

Of note however, within the further disease course, 76% (*n* = 22) of the patients in the PEX group and only 58% (*n* = 64) patients in the SOC group developed a maximal HE ≥ 2 (*p* = 0.021). Although maximal grade of HE did not differ between the groups (*p* = 0.145), there was a significant higher progress rate to a higher grade of HE in the PEX group (*p* = 0.008) (Supplemental Table 1).

### Use and procedural characteristics of therapeutic plasma exchange

PEX was performed in a median (IQR) of 2 (1–3) days from hospital admission (Table 2). A median (IQR) of 3 (2−3) PEX sessions (mean +/− SD: 2.6 +/− 1.1) were conducted. PEX was used once daily until clinical recovery or until liver transplantation or death. In detail, a singular PEX session was used in five patients, two sessions in seven patients, three sessions in 13 patients, four sessions in three patients and finally six sessions in one patient. Median plasma volume, exchanged in a singular PEX session, was 4000 (3460–8000) ml, accounting for 1.7 (1.3–3.3) times the individual patient plasma volume. As replacement fluid, only donor FFP was used in all PEX procedures.

**Table 2:** Procedural characteristics of therapeutic plasma exchange (PEX) (*n* = 29)

Time difference hospital admission to PEX - days (median, [IQR])	2 [1–3]
Total number of performed PEX procedures - no (median, [IQR])	3 [2–3]
Plasma volume exchanged in singular PEX - ml (median, [IQR])	4000 [3460–8000]
Plasma volume exchanged in singular PEX - x IPV (median, [IQR])	1.7 (1.3–3.3)
Replacement fluid: fresh frozen plasma - no (%)	29 (100)

IPV – Individual patient´s plasma volume, PEX – Therapeutic Plasma Exchange

Given are procedural characteristics of therapeutic plasma exchange (PEX) as it was performed in the PEX group. Values are presented as median (25% to 75% interquartile range [IQR]) or if categorical as numbers and percentages

The following side effects were reported in association with PEX (% of the 29 PEX patients): hypo/hypercalcemia (28%), thrombocytopenia (21%), allergic reactions and hemodynamic instability (17% each), volume overload (10%), alkalosis (7%), and transfusion-related lung injury (TRALI, 3%). PEX application was stopped in only one patient (3%) due to side effects (TRALI) after two applications. All other PEX applications were continued until death, transplantation or recovery.

### Primary outcome

Within 28 days after fulfillment of ALF criteria, of the 111 patients included, 53 (47.7%) were listed for LTX, 35 (31.5%) eventually received a LTX and 29 (26.1%) patients died, among those 22 without receiving a transplant and seven following LTX.

**Fig. 2:**
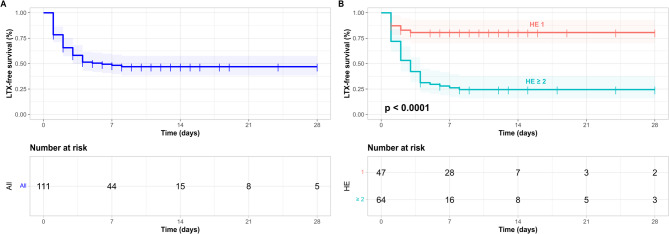
Liver-transplant-free survival in the entire patient cohort as well as stratified for grade of hepatic encephalopathy. Liver-transplant (LTX)-free survival following fulfillment of acute liver failure (ALF) criteria is shown as Kaplan-Meier graphs for all patients (**A**), and stratified by the grade of maximum hepatic encephalopathy (HE) (**B**) for a 28-day follow-up. p-values < 0.05 were considered significant

28-day LTX-free survival was 48.7% (*n* = 54/111) in the entire cohort (Fig. 2 A) However, LTX-free-survival was markedly different in patients, who experienced a HE with maximal grade 1 (*n* = 47) compared to those experiencing HE ≥ 2 (*n* = 64) (Fig. 2B). While LTX-free-survival was 80.9% (*n* = 38/47) in those with HE grade 1, it was only 25% (*n* = 16/64) in patients with HE ≥ 2 (*p* < 0.001) (Fig. 2B).

To further analyze whether PEX was used more frequently in recent years, we stratified patients by year comparing SOC and PEX (Supplementary Figure 1 A, Supplementary Figure 2 (interactive))**.** Numerically, in the time period of 2013–2018 three out of 35 patients received at least one PEX session (9%), whereas an increase in PEX frequency is seen for the time period of 2019–2024 (26 out of 76 patients received at least one PEX session (34%) (*p* = 0.005). This effect was even more pronounced if only PEX- centers were analyzed (*p* < 0.001) (Suppl. Figure 1B). Further analyses of different application rates of PEX in different centers, where at least one PEX was performed, did not show any significant differences between these centers (*p* = 0.7) (Supplementary Figure 1 C).

Most patients were included at high recruitment centers (three and more patients included). The LTX-free survival was numerically slightly higher in low recruitment centers (9 patients (60.0%) vs. 45 patients (46.8%)) without statistically significant difference (log-rank *p* = 0.47, Gehan-Breslow *p* = 0.46). Additionally, no significant difference was observed on LTX-free survival by center recruitment on univariate Cox-regression (HR 1.33, 95% CI: 0.57–3.11, *p* = 0.504).

### Primary outcome in subcohorts

Since incidence of maximal HE ≥ 2 during hospital stay was significantly higher in the PEX compared to the SOC cohort (see above), in the following, LTX-free survival was analyzed and compared between SOC and PEX groups both for the entire cohort (*n* = 111) as well as for patients with HE ≥ 2 (*n* = 64).

**Fig. 3:**
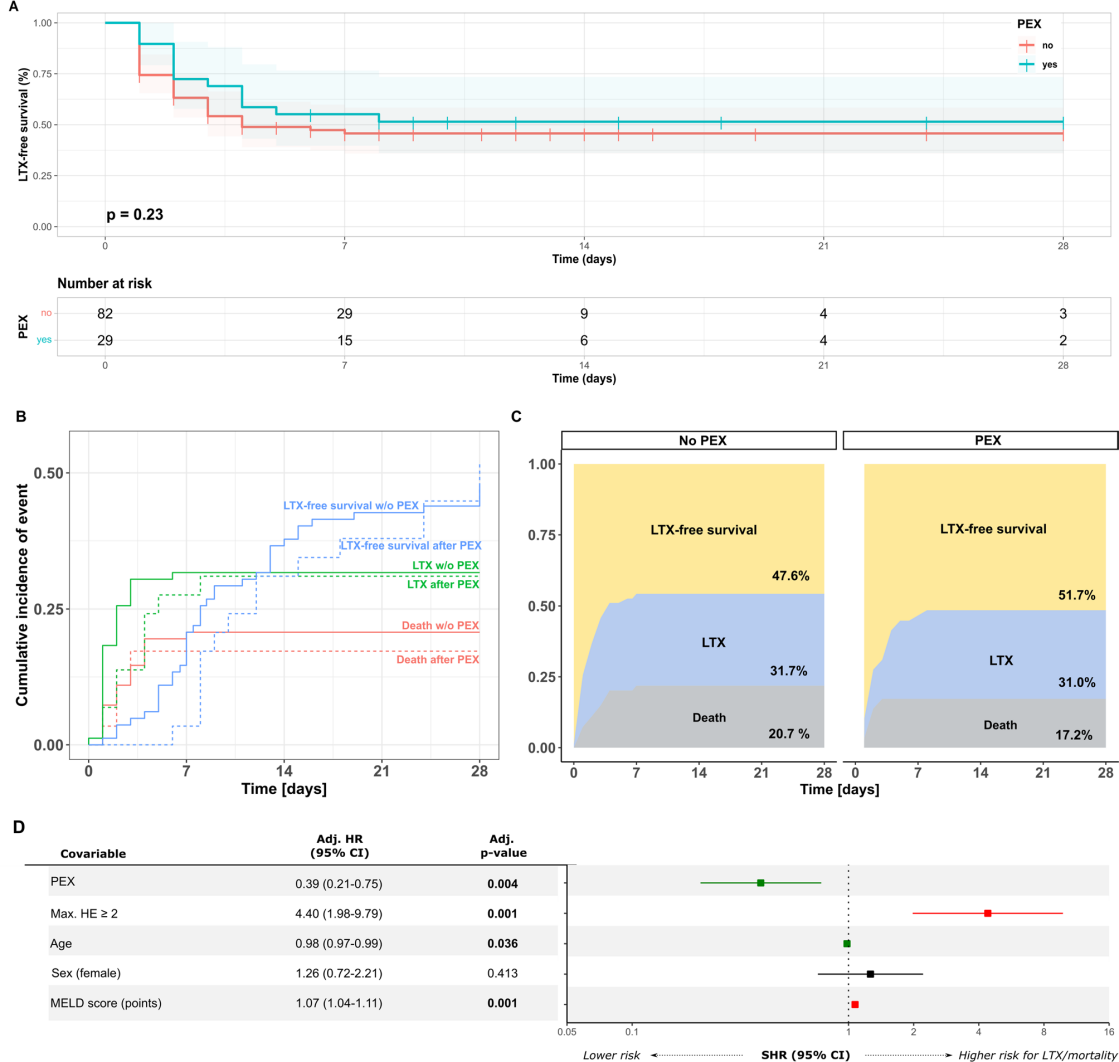
Influence of therapeutic plasma exchange on liver-transplant-free survival in the entire patient cohort. Liver-transplant (LTX)-free survival following fulfillment of acute liver failure (ALF) criteria is shown as Kaplan-Meier graphs for patients receiving standard of care (SOC) only and those receiving additional therapeutic plasma exchange (PEX) (**A**). Cumulative incidence function for LTX and death stratified by chosen therapeutic strategy (SOC vs. PEX) (**B**) and a multistate comparison of the cumulative incidence of LTX and death (**C**), are shown demonstrating comparable incidences of LTX and death in patients receiving SOC and PEX. Cumulative incidence function and multistate comparisons visualize the first occurring competing event of LTX or death, respectively. A corresponding multivariate cox-regression with hazard ratios (HR) and 95% confidence intervals (CIs) is shown as a forest plot with corresponding table (**D**). p-values < 0.05 were considered significant

28-day LTX-free survival was 47.6% (*n* = 39/82) in the SOC group and 51.7% in the PEX group (*n* = 15/29) for the entire cohort (Gehan-Breslow-Wilcoxon *p* = 0.230, Log-Rank *p* = 0.384, Fig. 3 A). A cumulative incidence function for LTX and death (competing risk) stratified by intervention (SOC vs. PEX) (Fig. 3B) and a multistate comparison of the cumulative incidence of LTX and death (Fig. 3C) demonstrate a numerically lower incidence of both LTX and death within 28 days in the PEX group. A multivariate cox-regression including PEX, max. HE ≥ 2, age, sex and MELD-Score at baseline, demonstrates that PEX therapy was independently associated with reduced risk of the combined endpoint liver transplantation or death within 28 days from inclusion (Hazard Ratio (HR) 0.39 (95% Confidence Interval (CI) 0.21–0.75, *p* = 0.004, Fig. 3D and Supplementary Table 2). In an additional competing-risk-regression analysis PEX however was not associated with increased LTX-free-survival in the entire cohort (Supplementary Table 2).

**Fig. 4:**
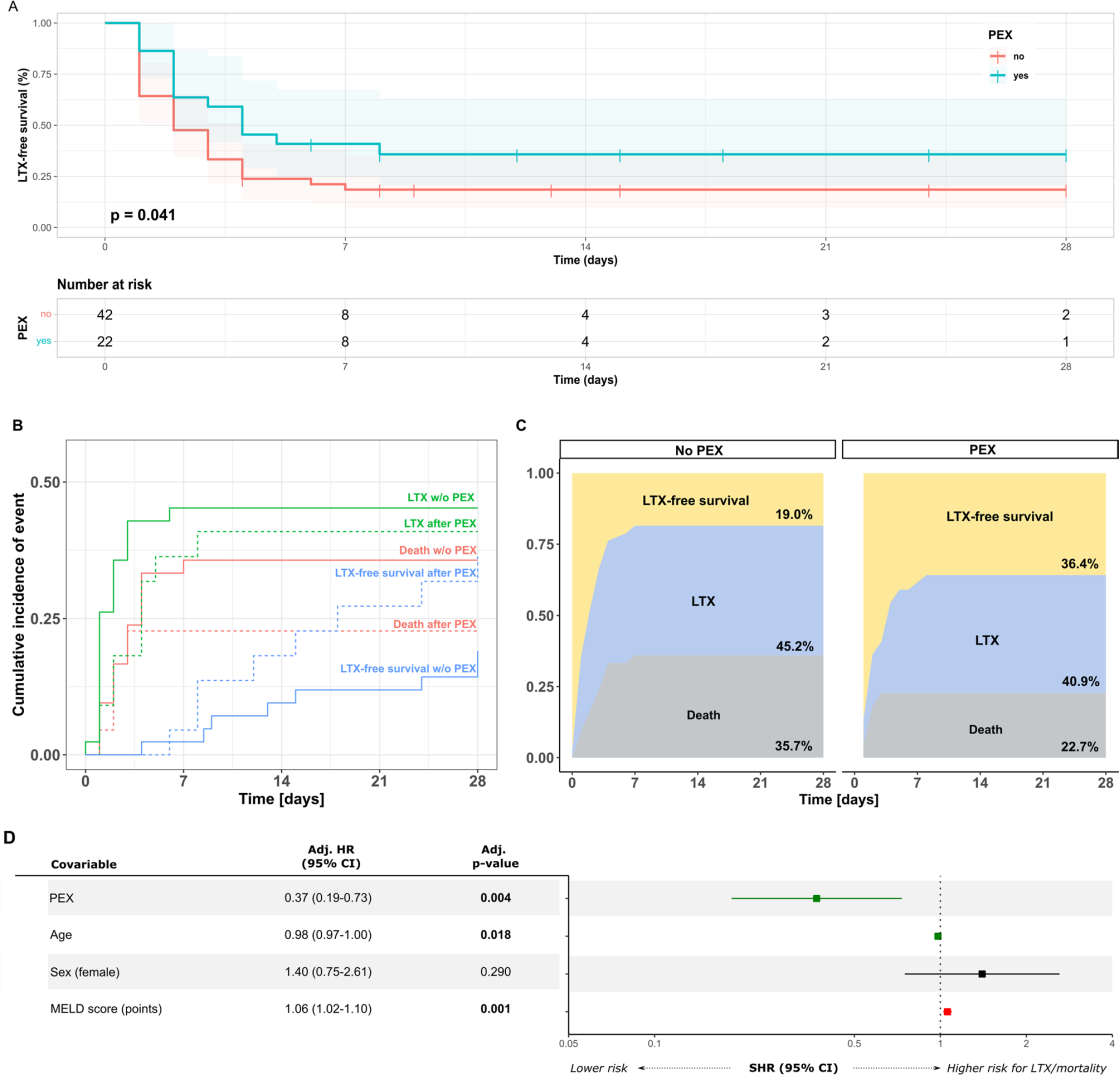
Influence of therapeutic plasma exchange on liver-transplant-free survival in patients with maximal hepatic encephalopathy grade ≥ 2. Liver-transplant (LTX)-free survival following fulfillment of acute liver failure (ALF) criteria is shown as Kaplan-Meier graphs for patients with maximal hepatic encephalopathy (HE) grade ≥ 2 receiving standard of care (SOC) only and those receiving additional therapeutic plasma exchange (PEX) (**A**). Cumulative incidence function for LTX and death stratified by chosen therapeutic strategy (SOC vs. PEX) (**B**) and a multistate comparison of the cumulative incidence of LTX and death (**C**), are shown demonstrating a lower combined incidence of LTX and death in patients with HE grade ≥ 2 receiving PEX. Cumulative incidence function and multistate comparisons visualize the first occurring competing event of LTX or death, respectively. A corresponding multivariate cox-regression with hazard ratios (HR) and 95% confidence intervals (CIs) is shown as a forest plot with corresponding table (**D**). p-values < 0.05 were considered significant

In the subgroup of patients with maximal HE ≥ 2, 28-day LTX-free survival was 19.1% (*n* = 8/42) in the SOC group, while it was 36.4% (*n* = 8/22) in patients receiving adjunctive PEX (Gehan-Breslow-Wilcoxon *p* = 0.041, Log-Rank *p* = 0.060, Fig. 4A). A cumulative incidence function for LTX and death (competing risk) stratified by intervention (SOC vs. PEX) (Fig. 4B) and a multistate comparison of the cumulative incidence of LTX and death (Fig. 4C) demonstrate a numerically lower incidence of both LTX and death within 28 days in the PEX group. PEX was independently associated with reduced risk of the combined endpoint death or liver transplantation within 28 days from inclusion when adjusted for covariables age, sex and MELD-Score at baseline in a multivariate cox-regression analysis (Hazard Ratio (HR) 0.37 (95% Confidence Interval (CI) 0.19–0.73, *p* = 0.004, Fig. 4D and Suppl. Table 3). Additional competing-risk-regression analysis confirmed that PEX was associated with increased LTX free-survival in patients with HE ≥ 2 (Suppl. Table 3).

In the subgroup of patients with maximal HE = 1, 28-day LTX-free survival was 77.5% (*n* = 31/40) in the SOC group, while it was 100% (*n* = 7/7) in patients receiving adjunctive PEX (Gehan-Breslow-Wilcoxon *p* = 0.176, Log-Rank *p* = 0.175, Suppl. Figure 3 A-B). Due to the low number of patients in the SOC- and no negative events in the PEX group no further multistate comparisons were performed for the subgroup with maximal HE = 1.

Visualization of LTX-free survival dependent on absolute PEX frequency showed no effect of PEX session frequency on LTX-free survival (Suppl. Figure 4 A). Univariate Cox-regression analysis testing the influence of absolute PEX frequency on LTX-free survival also did not reveal a significant effect (HR 0.797, 95% CI: 0.493–1.288, *p* = 0.353). No significant difference in applied PEX sessions was seen between patients with HE grade 1 and patients with HE grade ≥ 2 (Suppl. Figure 4B).

### Primary outcome in a propensity score matching analysis

**Fig. 5:**
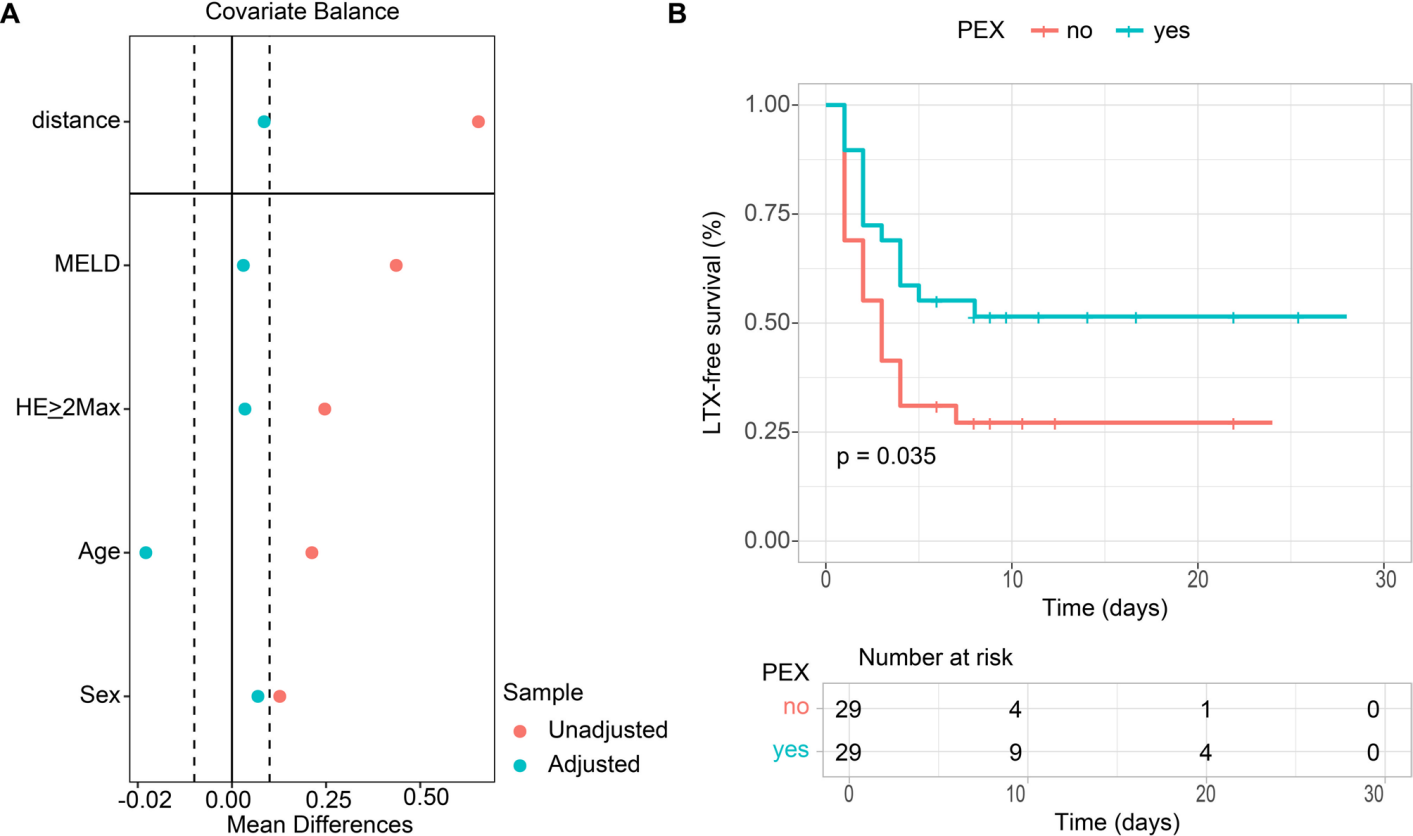
Influence of therapeutic plasma exchange on liver-transplant-free survival in a propensity-score-matching analysis. A love plot showing the variables used for the propensity score matching (PSM) and their balance before and after PSM. All the variables, except for age, achieved proper balance (standardized mean difference < 0.1) after matching (**A**). Kaplan–Meier curve with Log-rank test for LTX-free survival of the cohort after PSM (**B**)

Additional 1:1 propensity score matching (PSM) of all 29 PEX patients with corresponding 29 SOC patients matching for the four variables Age, Sex, MELD score at baseline and maximal HE grade ≥ 2 was performed (Fig. 5A). The results of this PSM revealed improved 28-day LTX-free survival in patients receiving adjunctive PEX compared to SOC. 28-day LTX-free survival was 28% (*n* = 8/29) in the SOC group and 52% in the PEX group (*n* = 15/29) (Gehan-Breslow *p* = 0.036; Log-Rank *p* = 0.035) (Fig. 5B).

### Secondary outcomes

**Fig. 6:**
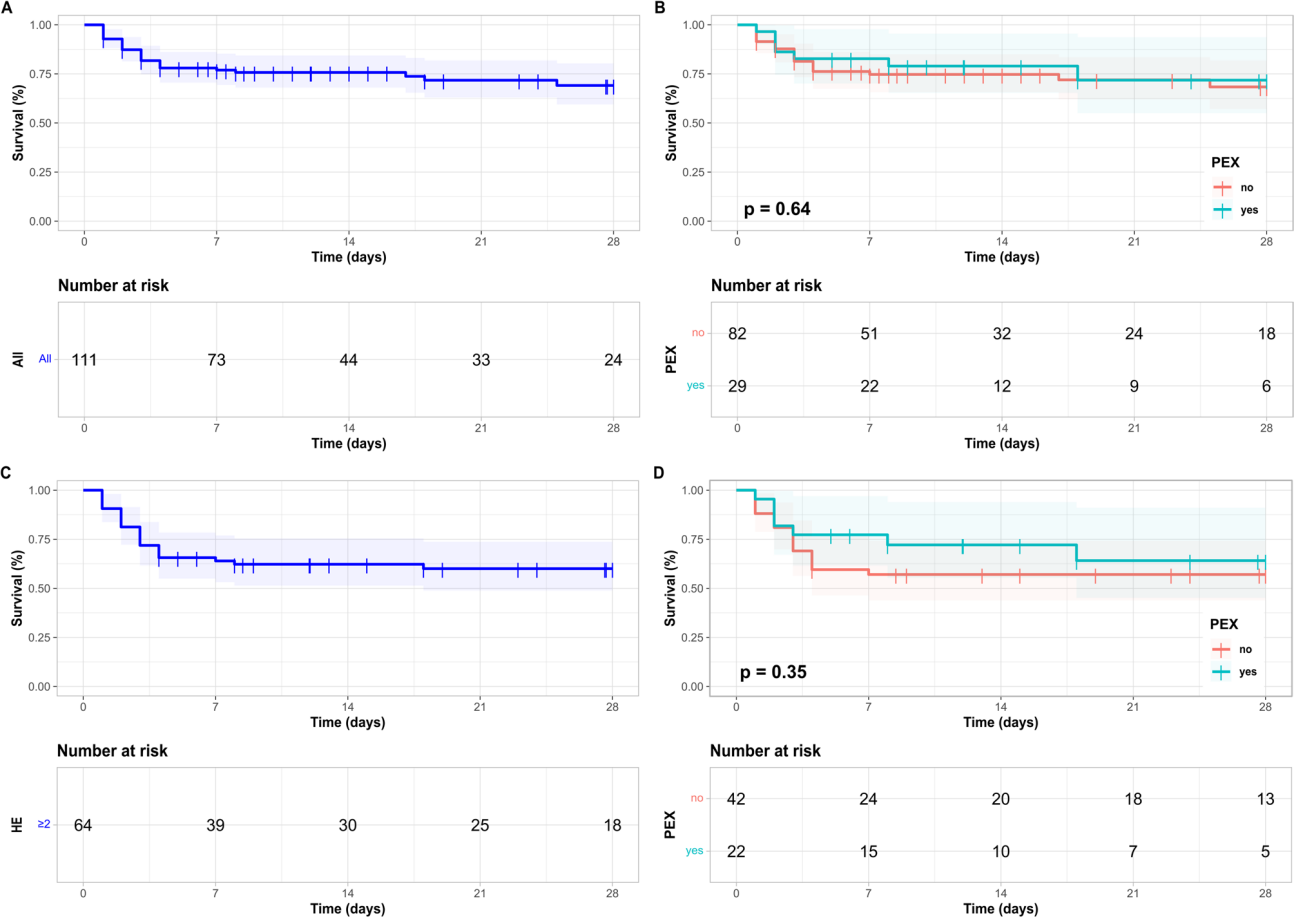
Overall survival in the entire patient cohort and in patients with hepatic encephalopathy grade ≥ 2. Overall survival (OS) for 28-days following fulfillment of acute liver failure (ALF) criteria is shown as Kaplan-Meier graphs for all patients (**A**) and for patients additionally stratified by therapeutic strategy (standard of care (SOC) vs. adjunctive therapeutic plasma exchange (PEX) (**B**) in the entire cohort. Further, OS is shown for the subgroup of patients with maximal hepatic encephalopathy (HE) grade ≥ 2 (**C**) and for these patients additionally stratified by therapeutic strategy (SOC) vs. PEX) (**D**). p-values < 0.05 were considered significant

Overall survival (OS) until day 28 was not different between SOC and PEX groups. OS in the entire cohort was 73.9% (*n* = 82/111) (Fig. 6A**)**, with 73.2% (*n* = 60/82) of patients in the SOC and 75.9% (*n* = 22/29) of patients in the PEX group surviving (Gehan-Breslow-Wilcoxon *p* = 0.64, Log-Rank *p* = 0.7, Fig. 6B) until day 28, respectively. OS in the cohort with HE ≥ 2 was 60.9% (*n* = 39/64) (Fig. 6C), with 57.1% (*n* = 24/42) of patients in the SOC and 68.2% (*n* = 15/22) of patients in the PEX group surviving (Gehan-Breslow-Wilcoxon *p* = 0.35, Log-Rank *p* = 0.41, Fig. 6D) until day 28, respectively. Following LTX, a total of seven patients still died within 28 days after study inclusion, among those two in the PEX and five in the SOC group. OS of patients with HE = 1 was similar in both treatment groups (90% (*n* = 36/40) vs. 100% (*n* = 7/7), Gehan-Breslow-Wilcoxon *p* = 0.476, Log-Rank *p* = 0.739) (Suppl. Figure 3 C-D).

OS until day 28 in the propensity score matched cohort was 67% (*n* = 39/58) with no significant difference between SOC (59%; *n* = 17/29) and PEX group (76%; *n* = 22/29) (Gehan-Breslow-Wilcoxon *p* = 0.094, Log-Rank *p* = 0.069).

Median (IQR) length of hospital stay (LOS) was 9 (5–24) days vs. 12 (8–27) days in the SOC and PEX group (*p* = 0.184), respectively. Incidence of AKI during hospital stay was 75.6% (*n* = 62/82) in the SOC- and 75.9% (*n* = 22/29) in the PEX group (*p* = 0.978). RRT was commenced in 43.9% (*n* = 36/82) of patients in the SOC- and 62.1% (*n* = 18/29) in the PEX group (*p* = 0.093). Invasive ventilation was needed in 47.6% (*n* = 39/82) of SOC and 48.3% (*n* = 14/29) of PEX patients (*p* = 0.947). 47.6% (*n* = 39/82) in the SOC- and 51.7% (*n* = 15/29) in the PEX group received vasopressor support. Likewise, no significant differences in these secondary outcomes were found between PEX and SOC patients in the sub-cohort of maximal HE above grade 1.

## Discussion

In this real-world, multi-center, multi-national study, we present the outcomes of the largest cohort of patients with AT-ALF to the present date. The results indicate that LTX-free survival of these patients, once ALF has developed, lies below 50% with a dramatic deterioration to only 25%, if HE exceeds grade 1. Adjunctive use of PEX was independently associated with increased LTX-free survival in patients with AT-ALF and HE grade ≥ 2. Additional PSM analysis, balancing for age, sex, baseline MELD and incidence of higher grade HE, also suggested improved LTX-free survival on patients receiving PEX. PEX was not associated with improved OS (with a trend towards better OS in the PSM analysis (*p* = 0.069)) or other secondary endpoints such as shorter LOS or lower incidence of AKI, need for RRT, invasive ventilation or vasopressor support during hospital stay.

Considering that ALF is already classified as a rare disease, AT-ALF-comprising less than 4% of its etiologies-should be regarded as an ultra-rare condition [17, 18]. In the United States Acute Liver Failure Study Group (US-ALFSG) registry from 01/1998 to 12/2014 among 2224 ALF cases, only thirteen AT-ALF patients were registered [27]. The most recent multicenter study from the UK, including 378 ALF patients, contained no AT-ALF cases [15]. Thus, this present study, identifying in a multi-national approach 111 patients with AT-ALF, represents by far the most concise report on AT-ALF to the present date. Beyond the focus on PEX, this study shows that antidote therapy with silibinin and ALF therapy with NAC was broadly applied as standard of care in more than 89% of patients.

PEX and liver assist devices have been applied in Amanita poisoning on a case-by-case basis for more than 20 years, but with the primary aim of detoxification and interruption of the enterohepatic circulation of the amatoxins [28–31]. Due to the high protein binding of amatoxins and the limited capacity to remove amatoxins from the bloodstream, these studies could not show a clear clinical benefit. In clear contrast, the current study analyzed PEX exclusively as therapy for manifest ALF and not as therapy of the poisoning itself.

Overall, 28-day LTX free-survival in the entire cohort was 48.7%, aligning well with previously reported LTX-free survival in AT-ALF (46%) [27] and ALF in general (45%) [15]. Likewise, an OS of 73.9% in this study was comparable to a more recently reported OS of 69% in undifferentiated ALF [15].

Of note, LTX-free survival dramatically decreased to only 25%, when patients developed HE grade 2 or higher, confirming the strong predictive value of higher grade HE in ALF [22] on inferior outcome also for AT-ALF. Utilization of PEX was not standardized in this investigation and although patients in both groups were largely comparable at baseline, PEX was used in a significantly higher proportion of patients, further deteriorating with HE ≥ 2 (76% vs. 58%). Correspondingly, progress to a higher HE grade was significantly more often observed in the PEX group, potentially representing higher disease severity in these patients. Likewise, a recent analysis from UK transplant centers found that PEX was preferentially initiated in ALF patients exhibiting markers of higher illness severity and organ failure [15]. Importantly Larsen et al., showing improved LTX-free survival in ALF with adjunctive PEX therapy, included only patients with HE ≥ 2 [7]. Therefore, in this study the primary endpoint LTX-free survival was reported both for the unselected entire cohort as well as for patients with only higher grade HE. LTX-free survival was comparable between PEX and SOC groups in the entire cohort. However, in the subgroup with higher grade HE, LTX-free survival was especially poor and below 20% in the SOC group while it was almost double as high in the PEX group. Both multivariate cox-regression- as well as competing-risk regression models suggested that PEX was independently associated with improved LTX-free survival in these patients.

While the RCT by Larsen et al. showed improved LTX-free-survival [7], a more recent observational study by Burke et al. suggested no difference in LTX-free survival by additive PEX in ALF patients [15]. Both studies included ALF from heterogeneous etiologies, while this present study only included AT-ALF patients. Extent of multi-organ dysfunction as well as need for vasopressor support, invasive ventilation and RRT was significantly higher in the cohort by Burke et al. compared to both the Larsen cohort and our cohort. This suggests that PEX was employed in the Burke cohort significantly later within the disease course with subsequent more manifest MOF, potentially then reducing the clinical efficacy of the intervention.

In the present study, the median time difference from ingestion to hospital admission was only two days, with PEX being initiated again only two days after admission. The rapid disease progression in AT-ALF as hyperacute ALF [1] and the initial overt gastrointestinal symptoms of amatoxin ingestion, then leading to earlier medical attendance, might have led to earlier utilization of PEX in this special patient cohort. Based on our real-world observation, we would hypothesize that hyperacute ALFs with necrosis of many liver cells with subsequent inflammatory cascades might benefit more from adjunctive PEX compared to subacute ALF e.g. caused by idiosyncratic drug induced liver injury (DILI). None of the available studies on PEX as therapy of ALF stratified patients according to the kinetic of ALF in a way as in the present study on just a monocausal ALF type with a homogenous hyperacute kinetic. Future studies on PEX on ALF might benefit from clearer subgroup analysis to tailoring the use of the expensive and resource intensive PEX to those ALF manifestations that benefit the most, e.g. AT-ALF with at least HE grade 2.

Larsen et al. used a high-volume (15% of body weight, approx. 8–12 L of exchange fluid) PEX regimen [7], while subsequent smaller studies suggested preserved efficacy of PEX in ALF despite employing normal exchange volume (1–2 plasma volumes, approx. 3–5 L) regimens [8, 13, 14]. In the present study, normal exchange-volume PEX was performed in all centers, corresponding well to the state of current practice reported recently by Burke et al. [15]. Th mean (+/− SD) number of total performed PEX sessions was well comparable between this present (2.6 +/− 1.1) and the Larsen study (2.4 +/− 0.8) [7], likewise the median (IQR) number (3 (2, 3)) was the same as observed in study by Burke et al. (3 (2, 3)) [15].

In the present study, side effects that were potentially associated with PEX applications were mostly absent or mild (*n* = 28/29, 97%), and PEX had to be discontinued due to side effects (TRALI) in only one patient (*n* = 1/29, 3%). There was no significant increase in PEX-associated side effects and adverse events compared to the SOC arms in the available RCTs [7, 14].

Our study has several important limitations. Due to the retrospective, real-world, multi-center design, variation in the general management of ALF as well as the specific decision for implementation of adjunctive PEX between different centers cannot be fully accounted for giving rise to significant selection bias. Inevitably, the observational nature of this study and potential unmeasured baseline differences between the SOC and PEX groups, introduces a significant potential for bias in analyzing clinical endpoints. A more recent retrospective multicenter study from the UK [15] showed how heterogeneous and infrequent PEX is applied even in high volume centers. The fact that only nine of the 25 (36%) study centers and in six of the 10 (60%) countries applied PEX in the present study underlines, that there is a high center bias. The bias towards higher HE grades in the PEX group could reflect, that the centers applied PEX guided by the landmark RCT published in 2016 [7], where HE at least grade 2 was the inclusion criteria. This is further underlined by the fact that PEX were just applied after 2016. The separate analysis of HE1 and HE2-4 in this present study was performed to reach the best comparability with the Larsen-RCT. However, an increase in PEX utilization could be noticed since the landmark Larsen-RCT in 2016 with no significant differences between PEX-centers. Finally, it is important to state that centers with low patient recruitment might still have a high experience in performing PEX (e.g. Barcelona) and different recruitment numbers are influenced by the overall number of intoxication cases based on geographical differences for growth of the amanita fungi. Of note, no difference in outcome between different PEX centers could be observed.

Adjustment for these differences was attempted in this study by separately analyzing the subgroup with higher grade HE and by the implementation of multivariate regression models. The unequal contribution of the centers in this study does not necessarily reflect their expertise but is more likely determined by the geographical region and the growing corresponding specific conditions for Amanita, e.g. the majority of centers (*n* = 21/25, 84%) are members of the ERN Rare Liver Reference network. Although, a RCT clearly would be highly desired to investigate the therapeutic value of PEX in AT-ALF, given the rarity of this specific ALF entity with large seasonal and regional differences, the feasibility of conducting such a RCT in AT-ALF appears to be low. Of note, the recruitment phase of the Larsen trial was already 12 years.

## Conclusions

In conclusion, this real-world study reports poor outcome in AT-ALF patients with higher grade HE and suggests that adjunctive PEX therapy might improve LTX-free survival in these patients with high grade hepatic encephalopathy (≥ grade 2). However, PEX was not associated with increased LTX-free-survival in patients with hepatic encephalopathy grade 1 and did not improve overall-survival or other secondary endpoints such as shorter length of hospital stay or lower incidence of acute kidney injury, need for renal-replacement therapy, invasive ventilation or vasopressor support.

## Electronic supplementary material

Supplementary Material 1

Supplementary Material 2

Supplementary Material 3

Supplementary Material 4

Supplementary Material 5

Supplementary Material 6

Supplementary Material 7

## Data Availability

The data generated and analyzed during the current study are included in this published article or available from the corresponding author on reasonable request.

## Abbreviations

ALF: Acute liver failure
AT-ALF: Amatoxin-related acute liver failure
ACLF: Acute-on chronic liver failure
AKI: Acute kidney injury
ALT: Alanine amino-transferase
AST: Aspartate amino-transferase
CI: Confidence Interval
DAMPs: Damage-associated molecular patterns
EASL: European Association for the Study of the Liver
FFP: Fresh frozen plasma
HE: Hepatic encephalopathy
HR: Hazard Ratio
INR: International normalized ratio
IQR: Interquartile Range
LOS: Length of hospital stay
LTX: Liver transplantation
MELD: Model for end-stage liver disease
MOF: Multi-organ failure
OS: Overall survival
RCT: Randomized controlled trial
RRT: Renal replacement therapy
SOFA: Sequential organ failure assessment
PEX: Therapeutic plasma exchange
US-ALFSG: United States Acute Liver Failure Study Group
