# Supplementary figures and images for: Therapeutic plasma exchange in amatoxin associated acute liver failure–results from the multi-center Amanita-PEX study
Source: Crit Care. 2025 Oct 30;29:458. doi: 10.1186/s13054-025-05560-y (PMC12573913; doi:10.1186/s13054-025-05560-y)

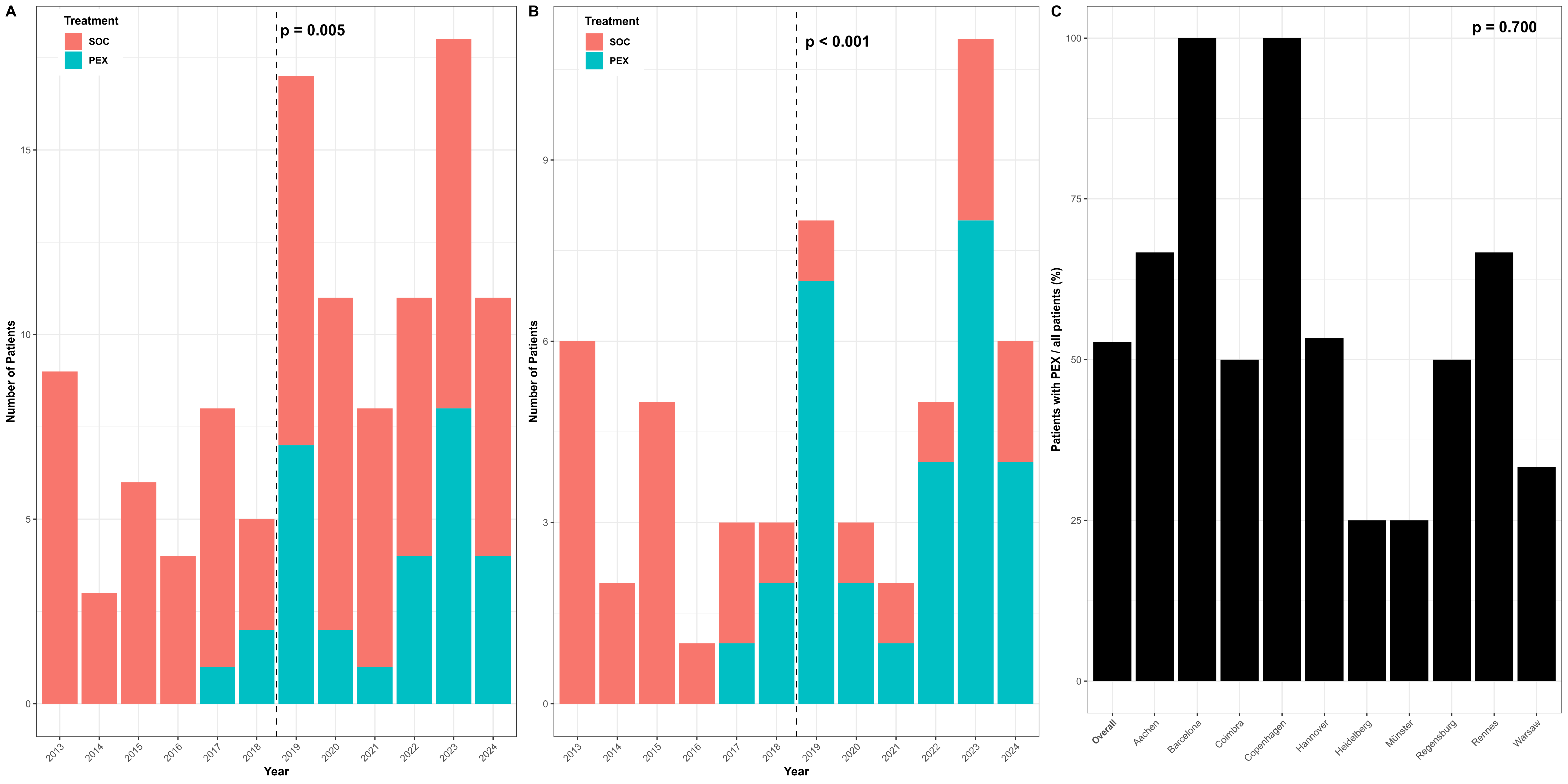

Supplement: Supplementary file 1 — Supplementary Material 1 [file 13054_2025_5560_MOESM1_ESM.png]

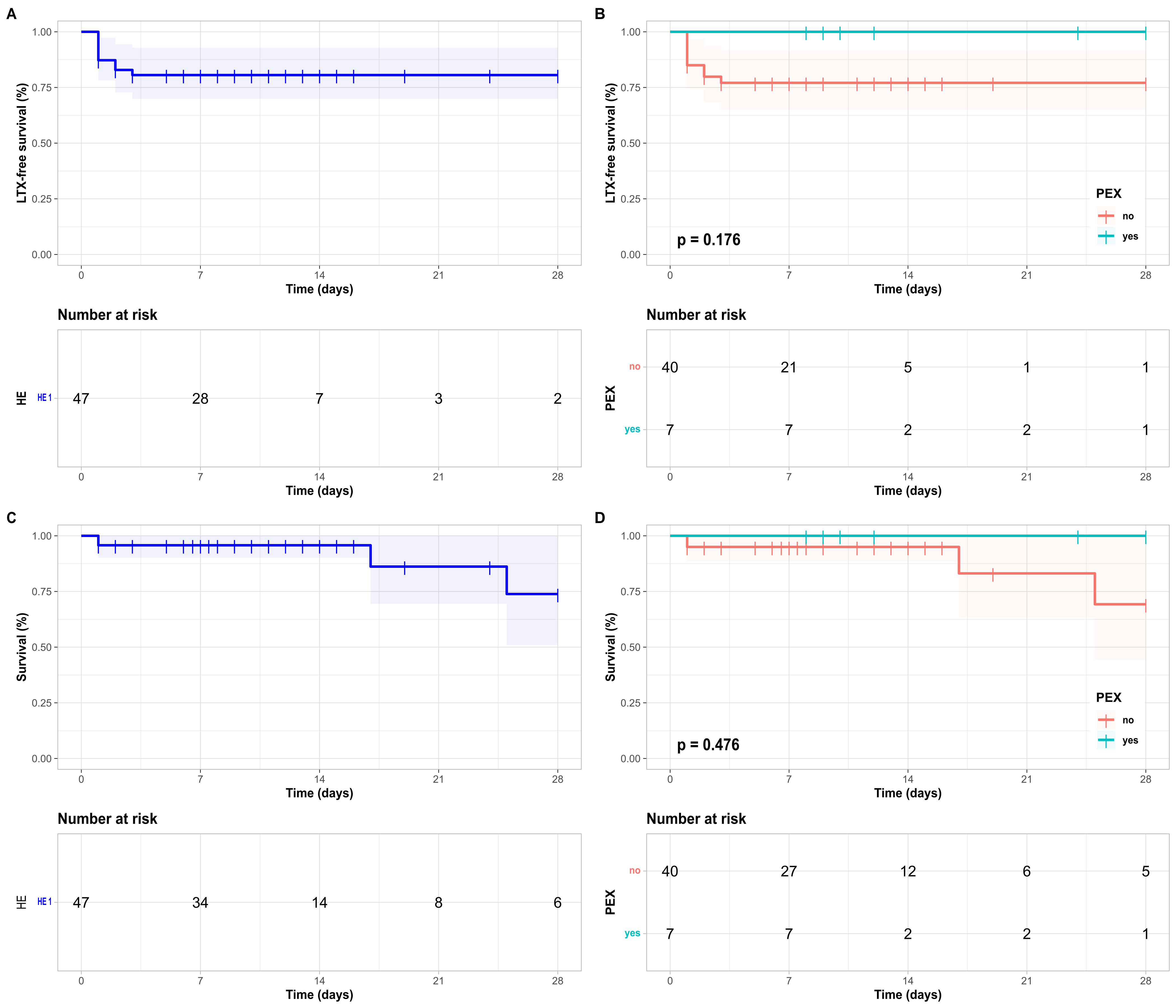

Supplement: Supplementary file 3 — Supplementary Material 3 [file 13054_2025_5560_MOESM3_ESM.png]

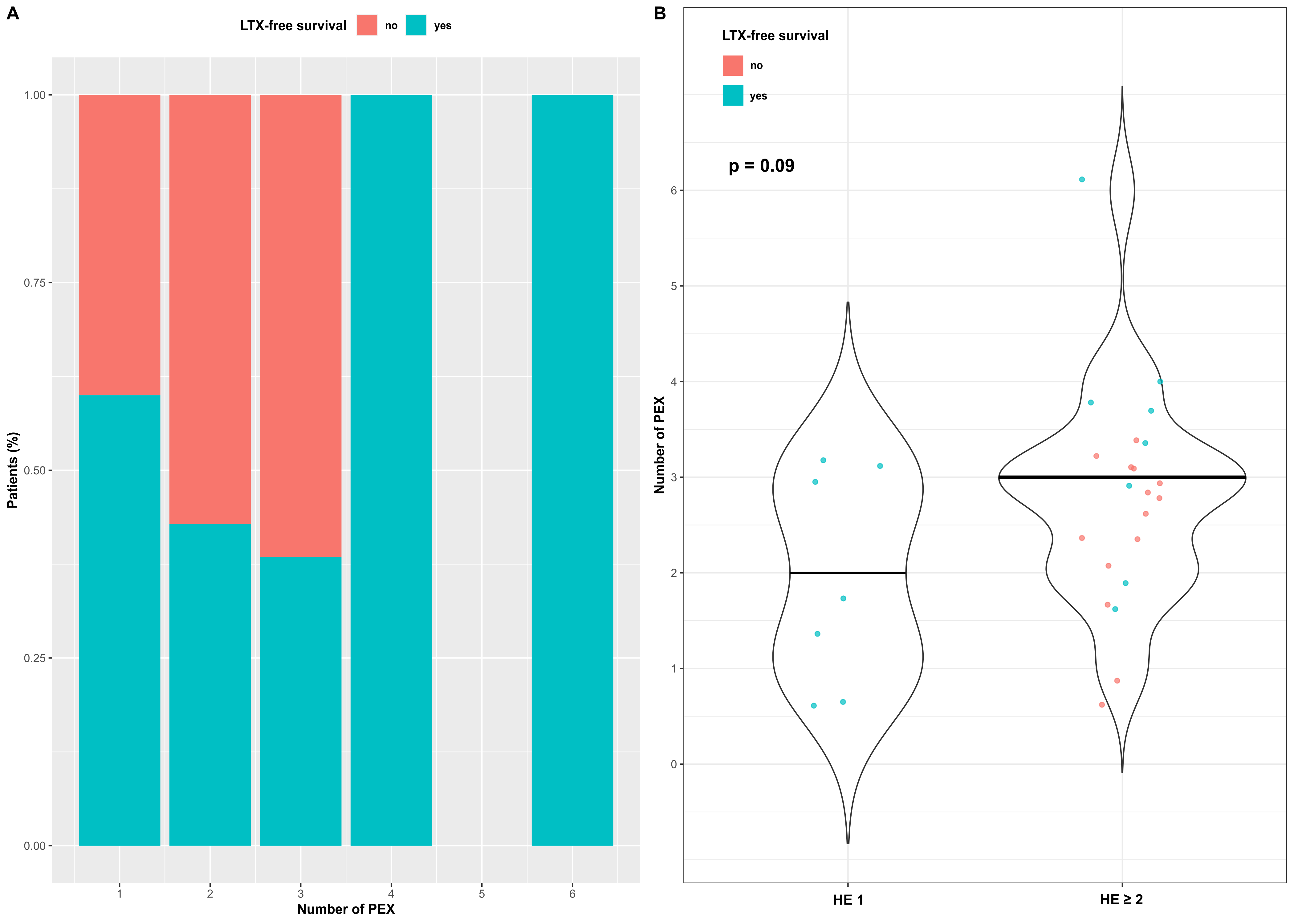

Supplement: Supplementary file 4 — Supplementary Material 4 [file 13054_2025_5560_MOESM4_ESM.png]
