# Supplementary material for: Therapeutic plasma exchange in amatoxin associated acute liver failure–results from the multi-center Amanita-PEX study
Source: Crit Care. 2025 Oct 30;29:458. doi: 10.1186/s13054-025-05560-y (PMC12573913; doi:10.1186/s13054-025-05560-y)
Supplement: Supplementary file 5 — Supplementary Material 5 [file 13054_2025_5560_MOESM5_ESM.docx]

| **Supplemental Table 1**: Longitudinal changes in hepatic encephalopathy grade divided by treatment group | | | | |
| --- | --- | --- | --- | --- |
| **Category** | **All** n=111 | **SOC** n=82 | **PEX** n=29 | **p** |
| HE grade at study inclusion |  |  |  | 0.618 |
| I | 60 (54.5%) | 44 (54.3%) | 16 (55.2%) |  |
| II | 23 (20.9%) | 15 (18.5%) | 8 (27.6%) |  |
| III | 20 (18.2%) | 16 (19.8%) | 4 (13.8%) |  |
| IV | 7 (6.4%) | 6 (7.4%) | 1 (3.4%) |  |
| Maximum grade of HE |  |  |  | 0.145 |
| I | 47 (42.3%) | 40 (48.8%) | 7 (24.1%) |  |
| II | 18 (16.2%) | 12 (14.6%) | 6 (20.7%) |  |
| III | 22 (19.8%) | 14 (17.1%) | 8 (27.6%) |  |
| IV | 24 (21.6%) | 16 (19.5%) | 8 (27.6%) |  |
| Progress of HE |  |  |  | **0.008** |
| No | 85 (76.6%) | 68 (82.9%) | 17 (58.6%) |  |
| Yes | 26 (23.4%) | 14 (17.1%) | 12 (41.4%) |  |

**Abbreviations:**

HE – Hepatic encephalopathy, PEX – Therapeutic Plasma Exchange, SOC – Standard of Care
