# Supplementary material for: Therapeutic plasma exchange in amatoxin associated acute liver failure–results from the multi-center Amanita-PEX study
Source: Crit Care. 2025 Oct 30;29:458. doi: 10.1186/s13054-025-05560-y (PMC12573913; doi:10.1186/s13054-025-05560-y)
Supplement: Supplementary file 6 — Supplementary Material 6 [file 13054_2025_5560_MOESM6_ESM.docx]

| **Suppl. Table 2:** Predictors of the primary endpoint in the entire patient cohort. | | | |
| --- | --- | --- | --- |
|  | **Multivariate Cox-Regression**  (Endpoint: death or liver transplantation within 28 days) | | |
| *Predictors* | *HR* | *CI* | *p* |
| PEX | 0.393 | 0.207 – 0.748 | **0.004** |
| Age - years | 0.984 | 0.970 – 0.999 | **0.036** |
| Sex | 1.263 | 0.722 – 2.210 | 0.413 |
| Hepatic encephalopathy grade ≥ 2 | 4.402 | 1.979 – 9.790 | **<0.001** |
| MELD-Score - points | 1.072 | 1.039 – 1.107 | **<0.001** |
|  | **Multivariate Competing risk regression**  (Endpoint: liver transplantation free survival within 28 days) | | |
| *Predictors* | *SHR* | *CI* | *p* |
| PEX | 1.367 | 0.827 – 2.258 | 0.220 |
| Hepatic encephalopathy grade ≥ 2 | 0.278 | 0.168 – 0.460 | **<0.001** |
| Age - years | 0.995 | 0.978 – 1.011 | 0.530 |
| Sex - female | 0.714 | 0.437 – 1.167 | 0.180 |
| MELD-Score - points | 0.933 | 0.908 – 0.959 | **<0.001** |

**Abbreviations:**

CI – Confidence Interval, HR – Hazard Ratio, MELD – Model of End-stage liver disease, PEX – Therapeutic Plasma Exchange, SHR – Subdistribution Hazard ratio
