# Supplementary material for: Therapeutic plasma exchange in amatoxin associated acute liver failure–results from the multi-center Amanita-PEX study
Source: Crit Care. 2025 Oct 30;29:458. doi: 10.1186/s13054-025-05560-y (PMC12573913; doi:10.1186/s13054-025-05560-y)
Supplement: Supplementary file 7 — Supplementary Material 7 [file 13054_2025_5560_MOESM7_ESM.docx]

| **Suppl. Table 3:** Predictors of the primary endpoint in patients with hepatic encephalopathy grade ≥ 2 | | | |
| --- | --- | --- | --- |
|  | **Multivariate Cox-Regression**  (Endpoint: death or liver transplantation within 28 days) | | |
| *Predictors* | *HR* | *CI* | *p* |
| PEX | 0.369 | 0.186 – 0.733 | **0.004** |
| Age | 0.981 | 0.966 – 0.997 | **0.018** |
| Gender | 1.401 | 0.751 – 2.613 | 0.290 |
| MELD | 1.059 | 1.023 – 1.097 | **0.001** |
|  | **Multivariate Competing risk regression**  (Endpoint: liver transplantation free survival within 28 days) | | |
| *Predictors* | *SHR* | *CI* | *p* |
| PEX | 5.460 | 2.049 – 14.549 | **<0.001** |
| Age - years | 1.029 | 1.003 – 1.056 | **0.029** |
| Sex - female | 0.338 | 0.107 – 1.066 | 0.064 |
| MELD-Score - points | 0.897 | 0.845 – 0.952 | **<0.001** |

**Abbreviations:**

CI – Confidence Interval, HR – Hazard Ratio, MELD – Model of End-stage liver disease, PEX – Therapeutic Plasma Exchange, SHR – Subdistribution Hazard ratio
